# Autonomous Water Quality Monitoring and Water Surface Cleaning for Unmanned Surface Vehicle

**DOI:** 10.3390/s21041102

**Published:** 2021-02-05

**Authors:** Hsing-Cheng Chang, Yu-Liang Hsu, San-Shan Hung, Guan-Ru Ou, Jia-Ron Wu, Chuan Hsu

**Affiliations:** Department of Automatic Control Engineering, Feng Chia University (FCU), No. 100, Wenhwa Road, Seatwen, Taichung 40724, Taiwan; hcchang@fcu.edu.tw (H.-C.C.); sshung@fcu.edu.tw (S.-S.H.); guanru0216.ece08g@nctu.edu.tw (G.-R.O.); g08420027@ccu.edu.tw (J.-R.W.); M0806986@o365.fcu.edu.tw (C.H.)

**Keywords:** unmanned surface vehicle, navigation, obstacle avoidance, water quality monitoring, water surface cleaning, remote navigation control

## Abstract

Water is one of the most precious resources. However, industrial development has made water pollution a critical problem today and thus water quality monitoring and surface cleaning are essential for water resource protection. In this study, we have used the sensor fusion technology as a basis to develop a multi-function unmanned surface vehicle (MF-USV) for obstacle avoidance, water-quality monitoring, and water surface cleaning. The MF-USV comprises a USV control unit, a locomotion module, a positioning module, an obstacle avoidance module, a water quality monitoring system, a water surface cleaning system, a communication module, a power module, and a remote human–machine interface. We equip the MF-USV with the following functions: (1) autonomous obstacle detection, avoidance, and navigation positioning, (2) water quality monitoring, sampling, and positioning, (3) water surface detection and cleaning, and (4) remote navigation control and real-time information display. The experimental results verified that when the floating garbage located in the visual angle ranged from −30° to 30° on the front of the MF-USV and the distances between the floating garbage and the MF-USV were 40 and 70 cm, the success rates of floating garbage detection are all 100%. When the distance between the floating garbage and the MF-USV was 130 cm and the floating garbage was located on the left side (15°~30°), left front side (0°~15°), front side (0°), right front side (0°~15°), and the right side (15°~30°), the success rates of the floating garbage collection were 70%, 92%, 95%, 95%, and 75%, respectively. Finally, the experimental results also verified that the applications of the MF-USV and relevant algorithms to obstacle avoidance, water quality monitoring, and water surface cleaning were effective.

## 1. Introduction

Covering approximately 71% of the Earth’s surface, oceans and rivers are home to billions of types of aquatic life, yet humans have not treated the aquatic environment in a friendly manner [1]. Because of human negligence, water pollution has accumulated for decades. The pollutants in water include industrial waste, sewage, radioactive materials, and plastic waste [2]. Therefore, it is necessary for researchers to develop unmanned surface vehicles (USVs), autonomous surface vehicles (ASVs), and autonomous underwater vehicles (AUVs) with lower development costs and higher resolution in time and space measurements for hydrographic measurement [3], path planning and navigation [4], obstacle avoidance [5], water quality monitoring [6], and water surface cleaning [7]. In terms of obstacle detection and avoidance, Villa et al. [5] designed a USV with a light detection and ranging (LiDAR) model to provide the obstacle information in Cartesian coordinates and proposed a line-of-sight (LOS)-based guidance, navigation, and control (GNC) algorithm to avoid obstacles. Wang et al. [8] used a navigation radar embedded on the USV to detect unknown obstacles and proposed an improved ant colony optimization (IACO) algorithm for avoiding collisions. Kim et al. [9] presented a deep learning-based obstacle segmentation algorithm, called Skip-ENet, to recognize obstacles in real time by using vision sensors embedded on the ASV. Steccanella et al. [10] utilized a U-net convolutional neural network (CNN) based method to segment the image taken by the camera placed on the low-cost ASV for waterline and obstacle detection. Guardeño et al. [11] used a LiDAR sensor mounted on the USV to detect static obstacles and developed a new autotuning environment for static obstacle avoidance (ATESOA) algorithm for reactive static obstacle avoidance.

Recently, a number of researchers have developed novel ASV or AUV systems for long-term continuous water quality monitoring and seawater sampling [6,12,13,14,15]. For example, Li et al. [12] implemented a water color remote sensing-oriented USV (WC-USV), which is composed of an unmanned surface vehicle platform with a ground control station, a data acquisition module, and a transmission module, to execute autonomous navigation and obstacle avoidance, water sample collection, water quality measurement, meteorological information measurement, and remote control tasks. Ferri et al. [6] developed a small-sized ASV, called a HydroNet ASV, to measure hydrocarbon and heavy metal concentrations by using custom-made miniaturized sensors and collect water samples. In addition, a VFH^+^ algorithm was utilized to avoid possible collisions based on the measurements of the laser scanner, sonar, and altimeter sensors placed on the HydroNet ASV. Madeo et al. [13] presented a low-cost USV, named the water environmental mobile observer (WeMo), to provide a modular array of sensors for measuring the pH value, oxidation-reduction parameter (ORP), salinity, and dissolved oxygen. Cao et al. [14] proposed an intelligent wide-area water quality monitoring and online analysis system to analyze the pH, total dissolved solids (TDS), and turbidity values for estimating the water quality level, which comprises an automatic cruise intelligent USV, a water quality monitoring system (WQMS), and a water quality analysis algorithm. Cryer et al. [15] presented an ASV, named the C-Worker 4 (CW4), equipped with the conductivity, temperature, depth (CTD), dissolved O_2_ (DO), pH, pCO_2_, nitrate, chlorophyll fluorescence, coloured dissolved organic matter (CDOM), turbidity, and dissolved organic carbon (DOC) sensors, to collect high-resolution measurements in shallow coastal environments. Autonomous vehicles expand the monitoring range in both time and space and increase the possibility of real-time marine and continuous weather monitoring. They have compatible computing systems for smart sampling (e.g., adaptive sampling [16] and optimal sampling [17]) with which not all current vessels are equipped. Additionally, the monitoring efficiency can be enhanced through coordination between autonomous vehicles with different features for different uses [18]. These advantages are the reasons why autonomous vehicles are widely employed in autonomous, complex, and distributed water quality monitoring systems. In particular, ASVs, capable of remote tasks and safe navigation, can be regarded as low-cost assets for water monitoring conducted using mobile sampling methods. They are also a cheaper and simpler monitoring apparatus compared with AUVs [6].

With the extended application of robots in recent years, many researchers have attempted to develop robots for cleaning regional environments [19,20,21,22]. However, those designed to clear garbage from water surfaces require further development [7]. Although semimanual garbage cleaning vessels are commonly used to collect plastic waste on water surfaces, they are large and thus restricted to use in rivers with larger areas or heavier waste. Therefore, using garbage cleaning vessels to remove low-density waste from small rivers is impractical. Moreover, such vessels are disadvantaged by their inability to identify which object to remove as well as by them potentially causing secondary pollution through discharged exhaust. Some intelligent cleaner robots have been developed for collecting floating garbage on water surfaces. For example, Kong et al. [7] developed an intelligent water surface cleaner robot (IWSCR) system with a vision module, a motion control module, and a grasping module, which utilized the YOLOv3 for garbage detection, the sliding-mode controller for vision-based tracking and steering, and the feasible grasping strategy for floating garbage grasping and collection. Wang et al. [23] proposed an autonomous robot to clean rubbish floating on a lake surface and utilized the Maneuvering Model Group (MMG) model approach to model hydrodynamics of the robot. Ruangpayoongsak et al. [24] developed a floating waste scooper robot for collecting plastic bottles floating on a water surface. Li et al. [25] designed a vision-based water surface garbage capture robot with the modified YOLOv3 algorithm for real-time garbage detection in dynamic aquatic environments.

In this study, we have designed a multi-function unmanned surface vehicle (MF-USV) with obstacle avoidance, water quality monitoring, and water surface cleaning functions. The MF-USV is a robotic system which is composed of a USV control unit, a locomotion module, a positioning module, an obstacle avoidance module, a water quality monitoring system, a water surface cleaning system, a communication module, a power module, and a remote human–machine interface. The MF-USV is equipped with the following functions: (1) autonomous obstacle detection, avoidance, and navigation positioning: navigating the vehicle by using the USV control unit to control the locomotion module, adopting the obstacle avoidance module to detect and avoid obstacles, and employing the positioning module to record the movement trajectory of the MF-USV. (2) Water quality monitoring, sampling, and positioning: detecting real-time water pH values, collecting water samples, and positioning the sampling points by using the water quality monitoring system. (3) Water surface detection and cleaning: detecting and collecting floating garbage on water surfaces by using the water surface cleaning system. (4) Remote navigation control and real-time information display: enabling monitors to navigate the MF-USV through the Bluetooth wireless module. In addition, the movement trajectory recorded by the global positioning system (GPS), water pH values, and positions of sampling points can be transmitted back through the communication module in real time and displayed on the remote human–machine interface. Finally, functions, types of sensors, and related obstacle avoidance, water quality monitoring, and water surface cleaning algorithms are information extracted from the existing published papers and summarized in Table 1 for straightforward comparison. Obviously, most of the existing USVs or ASVs focus on only one function of obstacle avoidance, water quality monitoring, and water surface cleaning. Hence, the purpose of this paper is to develop a MF-USV which can perform the obstacle avoidance, water quality monitoring, and water surface cleaning functions simultaneously.

The rest of this paper is organized as follows. In Section 2, we introduce the prototype design of the proposed USV. The water quality monitoring and water surface cleaning methods are then described in Section 3 and Section 4, respectively. The experimental results are presented in Section 5. Finally, conclusions are given in Section 6.

## 2. Prototype Design of Multi-Function Unmanned Surface Vehicle (MF-USV)

**Figure 1:**
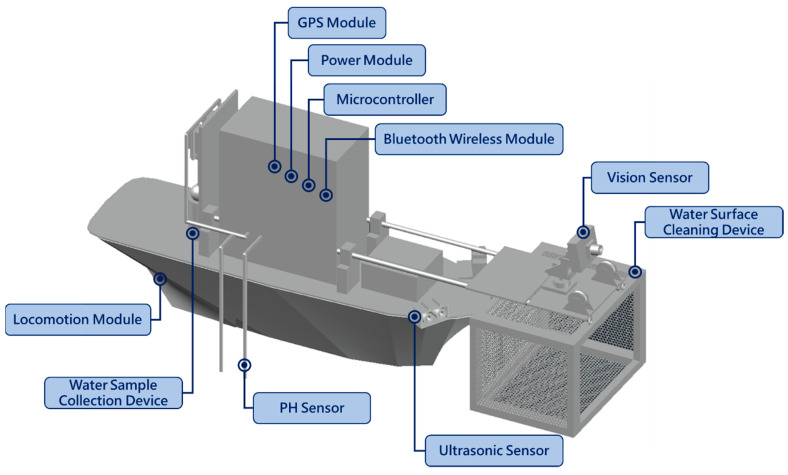
Overall view of the positions of all components inside the MF-USV.

**Figure 2:**
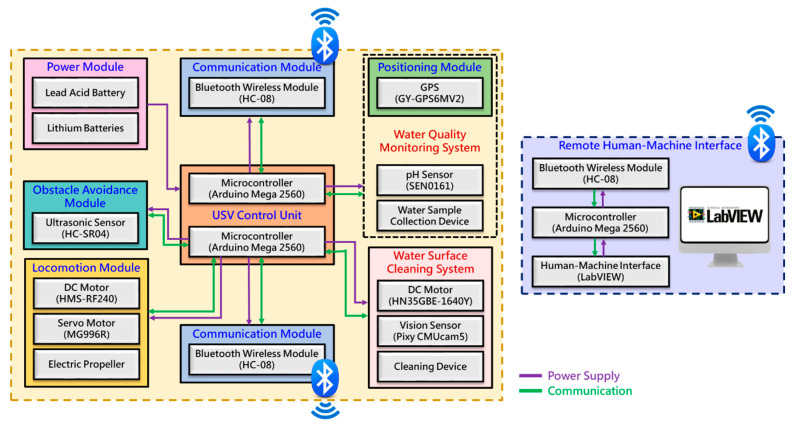
System architecture of the MF-USV.

The overall architecture design of the proposed MF-USV is shown in Figure 1. The MF-USV weights 3.025 kg, and its dimension is 0.74 m (length) × 0.3 m (width) × 0.28 m (height). The draft of the USV is 0.15 m and its maximum cruise speed is 0.15 m/s. The MF-USV architecture is composed of: (1) a main control unit, (2) a locomotion module, (3) a positioning module, (4) an obstacle avoidance module, (5) a water quality monitoring system, (6) a water surface cleaning system, (7) a communication module, (8) a power module, and (9) a remote human–machine interface, as shown in Figure 2. The different modules of the proposed MF-USV are detailed in Table 2.

### 2.1. USV Main Control Unit

The main control unit used in the MF-USV are two Arduino MEGA 2560 microcontrollers which are based on an AVR® 8-Bit Microcontroller at 16 MHz. The first Arduino MEGA 2560 microcontroller is responsible for locomotion, obstacle avoidance, and water surface cleaning tasks, while the second one is responsible for navigation positioning and water quality monitoring tasks. The MF-USV control unit is directly connected to the locomotion module, positioning module, obstacle avoidance module, water quality monitoring system, water surface cleaning system, communication module, and power module. Moreover, the MF-USV can transmit the multi-sensing data to the remote human–machine interface through two Bluetooth wireless modules for monitoring the movement trajectory of the MF-USV, the water pH values, and the water sampling positions.

### 2.2. Locomotion Module

**Figure 3:**
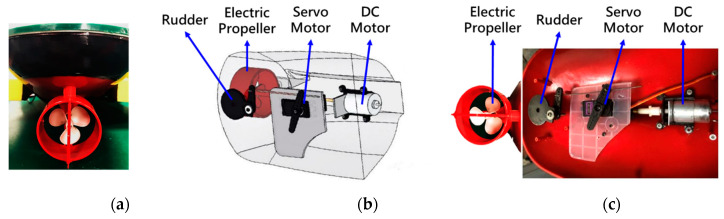
(**a**) Electric propeller and rudder. (**b**) Computer aided design (CAD) model of the locomotion module. (**c**) Locomotion module mounted on the MF-USV.

The locomotion module mounted on the MF-USV is composed of an electric propeller controlled by a direct current (DC) motor (HMS-RF-240) and a rudder controlled by a servo motor (MG996R), as shown in Figure 3. The DC and servo motors are both driven by a motor drive module (L298N) through the Arduino MEGA 2560 microcontroller. The DC motor connects with the electric propeller through the coupling for controlling the propulsion speed. The servo motor controls the steering blade connected to the rudder for controlling the propulsion direction.

### 2.3. Positioning Module

The positioning module mounted on the MF-USV is the GY-GPS6MV2 GPS Module (as shown in Table 3), which is used to generate the movement trajectory of the MF-USV during the voyage and record the position coordinates of the water sampling points. The GPS module utilizes the NEO-6M GPS receiver manufactured by U-Blox to provide time-to-first-fix (TTFF) acquisition of less than 1 second, which is composed of an antennae and a radio frequency (RF) satellite signal receiver, an interface device equipped with a microprocessor, and a base band processor for processing GPS signal. The Arduino MEGA 2560 microcontroller can read GPS coordinates of the MF-USV and water sampling points through the UART protocol and then send them to the remote human–machine interface through the communication module in real time. For our propose, we used the Recommended Minimum Specific GPS/TRANSIT Data ($GPRMC) output sentence to extract the travel time and local coordinates (latitude and longitude).

### 2.4. Obstacle Avoidance Module

**Figure 4:**
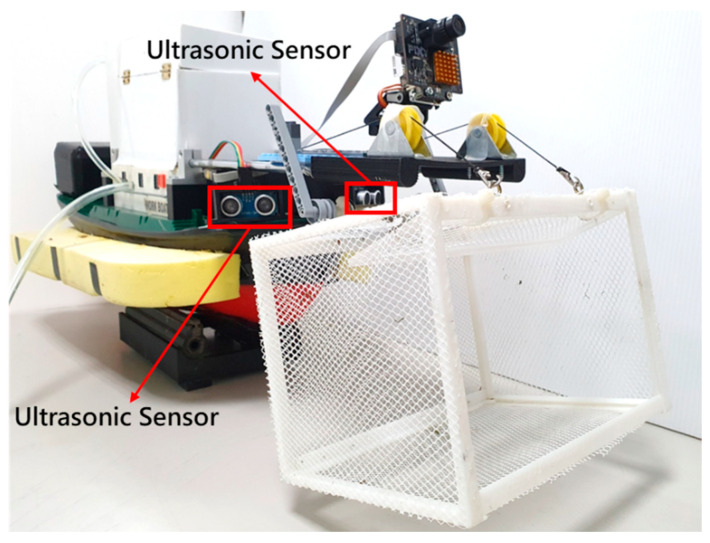
Ultrasonic sensors mounted on the MF-USV.

**Figure 5:**
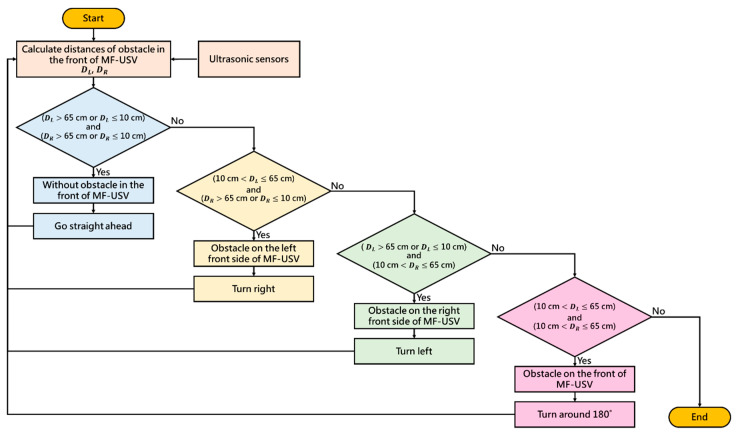
Threshold-based obstacle avoidance algorithm.

The obstacle avoidance module used in this paper is composed of two HC-SR04 ultrasonic sensors (as shown in Table 3), which are mounted on the left and right front of the MF-USV and provide a sensor detecting 15 degrees in front of it, as shown in Figure 4. The Arduino MEGA 2560 microcontroller can obtain the distance between the vehicle and obstacles in real time, which are detected by the ultrasonic sensors. Once the distance is greater or less than the threshold values, the MF-USV executes a threshold-based obstacle avoidance algorithm to avoid obstacle collision. The proposed threshold-based obstacle avoidance algorithm is shown in Figure 5. The first of the threshold-based algorithms calculates the distances of the obstacle in the left and right front of the vehicle (*D_L_* and *D_R_*) using the ultrasonic sensors mounted on the left and right front of the MF-USV. Subsequently, there are four situations for obstacle collision avoidance: (1) go straight ahead: when ($D_{L}>$ 65 cm or $D_{L}\leq$ 10 cm) and ($D_{R}>$ 65 cm or $D_{R}\leq$ 10 cm), the algorithm determines that there is no any obstacle on the front of the vehicle, and then the MF-USV goes straight ahead. (2) Turn right: when (10 cm $<D_{L}\leq$ 65 cm) and ($D_{R}>$ 65 cm or $D_{R}\leq$ 10 cm), the algorithm determines that there is an obstacle on the left front side of the vehicle, and then the MF-USV drives the servo motor (MG996R) in the locomotion module to control the rudder to turn right. (3) Turn left: when ($D_{L}>$ 65 cm or $D_{L}\leq$ 10 cm) and (10 cm $<D_{R}\leq$ 65 cm), the algorithm determines that there is an obstacle on the right front side of the vehicle, and then the MF-USV drives the servo motor (MG996R) in the locomotion module to control the rudder to turn left. (4) Turn around: when (10 cm $<D_{L}\leq$ 65 cm) and (10 cm $<D_{R}\leq$ 65 cm), the algorithm determines that there is an obstacle on the front of the vehicle, and then the MF-USV drives the servo motor (MG996R) in the locomotion module to control the rudder to turn around 180°.

### 2.5. Power Module and Communication Module

The MF-USV is powered by a 6 V lead-acid battery with a nominal capacity of 2.3 ampere-hour (Ah). The communication module includes two HC-08 Bluetooth wireless modules, which have low-power data transmission property and are operated in the V4.0 BLE Bluetooth protocol. The Arduino MEGA 2560 microcontroller embedded on the MF-USV transmits the GPS coordinates of the MF-USV and water sampling points, and the water pH values in different water sampling points to the remote human–machine interface through the Bluetooth wireless modules.

### 2.6. Remote Human–Machine Interface

**Figure 6:**
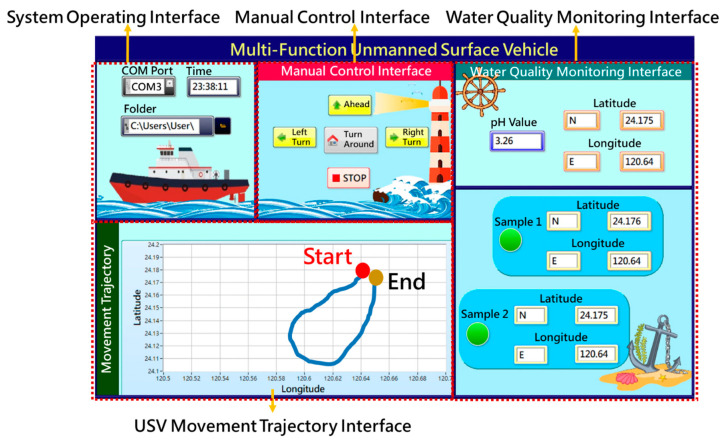
Remote human–machine interface.

The remote human-machine interface shown in Figure 6 is developed with the LabVIEW graphical programming environment, which can receive the GPS coordinates of the MF-USV and water sampling points, and the water pH values in different water sampling points from the MF-USV through the Bluetooth wireless modules. The remote human-machine interface is divided into the following operation interfaces: (1) system operating interface: the user can choose a series COM port to receive the measurement signals and set a storage path for storing them. In addition, the travel time of the MF-USV is also displayed. (2) Manual control interface: the user can manually control the MF-USV to go ahead, turn left, turn right, and turn around, respectively. In addition, the user can click the stop button to stop the MF-USV sailing. (3) Movement trajectory interface: this interface can display the movement trajectory during sailing of the MF-USV in real time via the position coordinates measured by the GPS positioning module. (4) Water quality monitoring interface: the pH value and local coordinate of the present position measured by the pH sensor and GPS module are displayed. In addition, the position coordinates of the water sampling points are also recorded and displayed in this interface.

## 3. Water Quality Monitoring System

**Figure 7:**
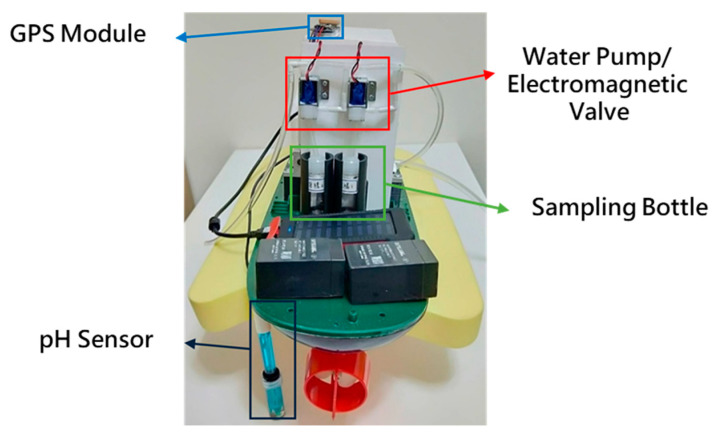
The position of the water quality monitoring system mounted on the MF-USV.

**Figure 8:**
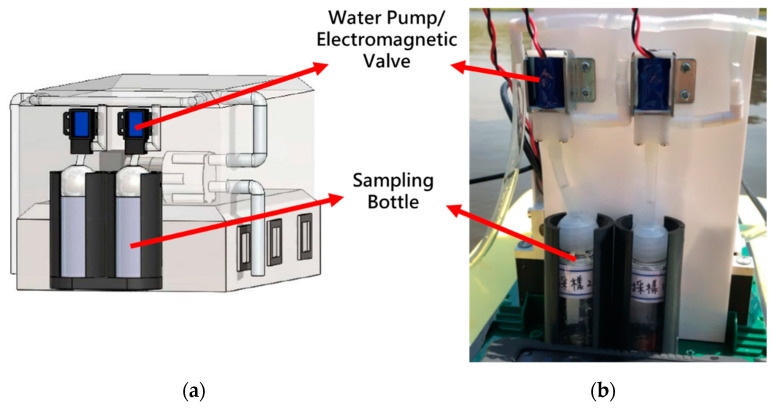
(**a**) CAD design model of the water sample collection device. (**b**) Water sample collection device mounted on the MF-USV.

**Figure 9:**
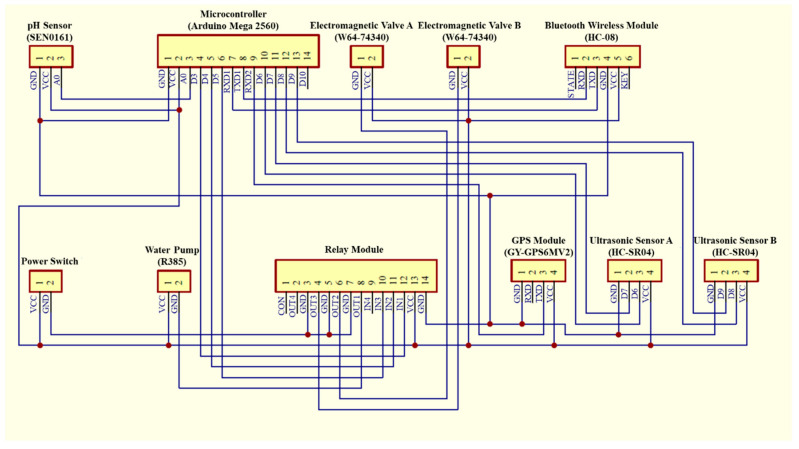
Circuit diagram of the water quality monitoring system.

**Figure 10:**
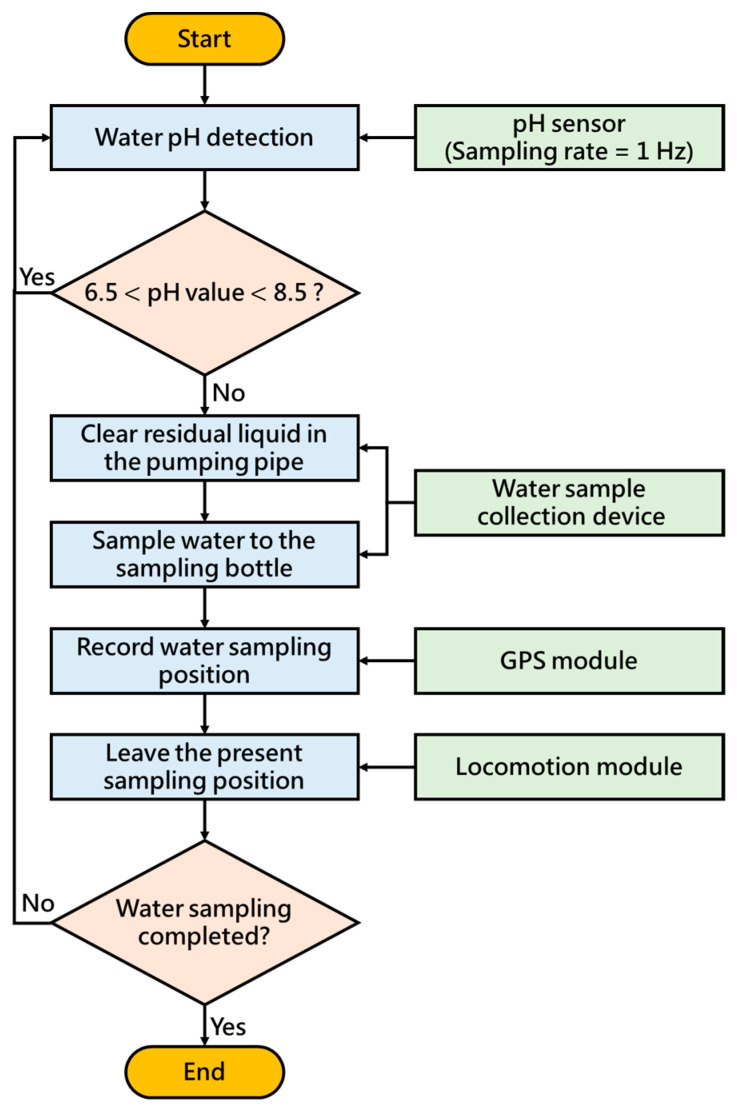
Water quality monitoring process.

The water quality monitoring system consists of a SEN0161 pH sensor (as shown in Table 3), a GPS module, and a water sample collection device, as shown in Figure 7. The water sample collection device is designed for collecting water samples, which is composed of a water pump (R385), two electromagnetic valves, and two sampling bottles, as shown in Figure 8. Figure 9 shows the circuit diagram of the water quality monitoring system mounted on the MF-USV. The water quality monitoring process is shown in Figure 10. The pH sensor mounted on the MF-USV is used to detect the pH value of the water during sailing, which is then sent to the remote human–machine interface in real time. According to the surface water classification and water quality standards of the Taiwan Environmental Protection Administration (EPA), the pH values of the water quality should be between 6.5 and 8.5, which comply with the class A terrestrial surface water quality standards [26]. Once the water pH value is higher than 8.5 or lower than 6.5, that means that the water on the present position is contaminated, the water pump is driven to extract the water for cleaning residual liquid in the pumping pipe. Subsequently, the water in the present position is sampled by the water pump through the pumping pipe. Then, the first electromagnetic valve is turn on for collecting the sampling water into the first sampling bottle. Simultaneously, the position coordinate of the present water sampling point measured by the GPS module is also recorded and sent to the remote human–machine interface through the Bluetooth wireless module. When the present water sampling task is finished, the water pump and the first electromagnetic valve are turned off, and the MF-USV leaves the present water sampling point to search the next contaminated water point. Once the pH value of the water in the next position is abnormal, the water sampling procedure is the same as the first sampling procedure. After the water quality monitoring task is completed, the MF-USV can automatically return to the initial location. The collected water samples are later analyzed in specialized laboratories.

## 4. Water Surface Cleaning System

**Figure 11:**
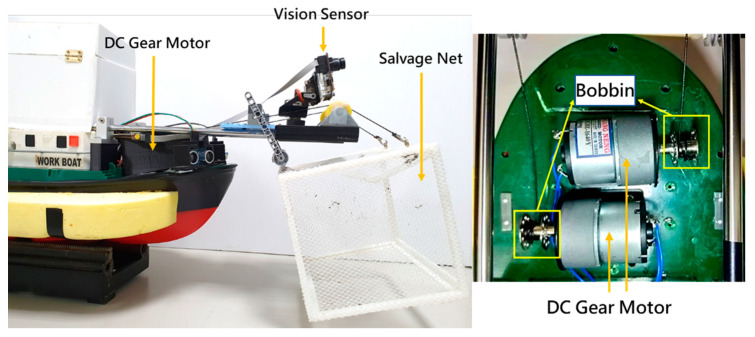
The position of the water surface cleaning system mounted on the MF-USV.

**Figure 12:**
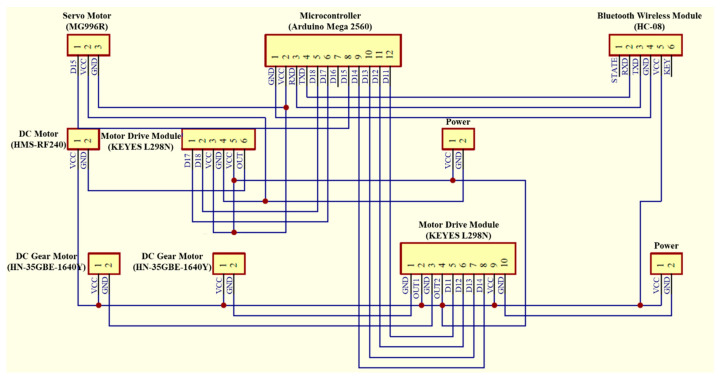
Circuit diagram of the water surface cleaning system and locomotion module.

The water surface cleaning system shown in Figure 11 consists of a Pixy CMUcam5 vision sensor (as shown in Table 3) and a water surface cleaning device. The Pixy CMUcam5 vision sensor is composed of a dual core processor NXP LPC4330 at 204MHz, an OmniVision image sensor, a 264K bytes RAM, and a 1M bytes Flash, with UART, SPI, I^2^C, and universal serial bus (USB) communication interfaces. The Pixy CMUcam5 vision sensor is utilized to detect objects by using a hue-based color filtering algorithm, which is called the color connected components (CCC) algorithm and implemented in the C/C++ programming language and on the processor NXP LPC4330. Since the hue-based color filtering methods are fast, efficient, and relatively robust, they are popular. The hue-based color filtering algorithm calculates the hue (color) and saturation of each red, green, and blue (RGB) pixel derived from the image sensor and uses these as the primary filtering parameters. The hue of the object remains basically unchanged under changes in lighting and exposure [27]. In this paper, a red color object is selected to represent the floating garbage to perform the water surface cleaning task because of its distinct colour for it can be easily distinguished from its surroundings. Hence, when the hue-based color filtering algorithm is utilized to detect the floating garbage, the MF-USV can detect it correctly. The water surface cleaning device is designed for cleaning and collecting the floating garbage, which is composed of two DC gear motors (HN35GBE-1640Y) and a salvage net. The DC gear motors are driven by a motor drive module (L298N) through the Arduino MEGA 2560 microcontroller, which are utilized to control the salvage net to rise and drop via the bobbins for collecting the floating garbage. Figure 12 shows the circuit diagram of the water surface cleaning system mounted on the MF-USV.

**Figure 13:**
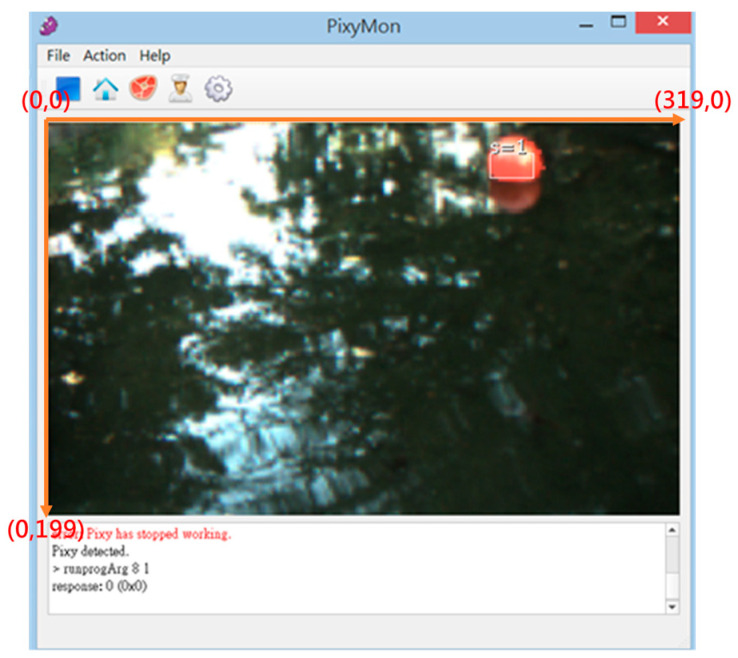
X and Y coordinates of the image extracted by the vision sensor.

**Figure 14:**
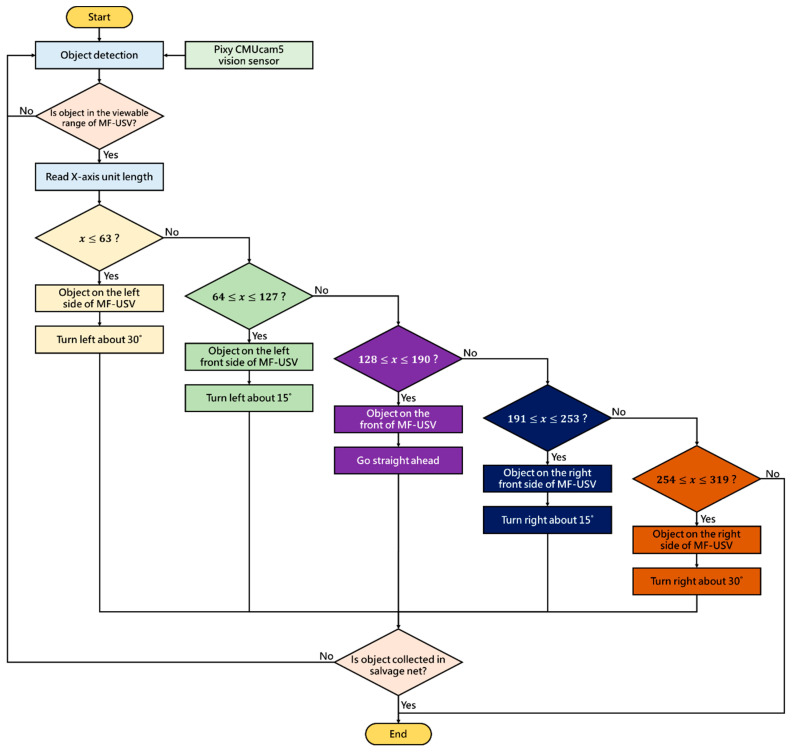
Water surface cleaning algorithm.

For the water surface cleaning task, we utilize the Pixy CMUcam5 vision sensor to detect the floating garbage (red color objects). When the vision sensor mounted on the front of the MF-USV detects floating garbage, the MF-USV can automatically track and collect the floating garbage through the water surface cleaning algorithm. The Pixy CMUcam5 vision sensor defines that the X and Y coordinates of the image ranged from 0 to 319 and from 0 to 199, as shown in Figure 13. The water surface cleaning algorithm is shown in Figure 14. First, with the water surface cleaning algorithm, the floating garbage is detected by the Pixy CMUcam5 vision sensor mounted on the front of the MF-USV, and its direction is determined based on the x coordinate of the image extracted by the vision sensor. Subsequently, there are five situations for the floating garbage cleaning and collection: (1) turn left about 30°: when 0 ≤ x ≤ 63, the algorithm determines that there is a floating garbage on the left side of the vehicle, and then the MF-USV drives the servo motor (MG996R) in the locomotion module to control the rudder to turn left about 30°. (2) Turn left about 15°: when 64 ≤ x ≤ 127, the algorithm determines that there is a floating garbage on the left front side of the vehicle, and then the MF-USV drives the servo motor (MG996R) in the locomotion module to control the rudder to turn left about 15°. (3) Go straight ahead: when 128 ≤ x ≤ 190, the algorithm determines that there is a floating garbage on the front side of the vehicle, and then the MF-USV goes straight ahead. (4) Turn right about 15°: when 191 ≤ x ≤ 253, the algorithm determines that there is a floating garbage on the right front side of the vehicle, and then the MF-USV drives the servo motor (MG996R) in the locomotion module to control the rudder to turn right about 15°. (5) Turn right about 30°: when 254 ≤ x ≤ 319, the algorithm determines that there is floating garbage on the right side of the vehicle, and then the MF-USV drives the servo motor (MG996R) in the locomotion module to control the rudder to turn right about 30°. In this way, the floating garbage will be on the front of the vehicle until that is collected by the water surface cleaning device. Finally, once the floating garbage is collected in the salvage net, the water surface cleaning task is completed.

## 5. Experimental Results

**Figure 15:**
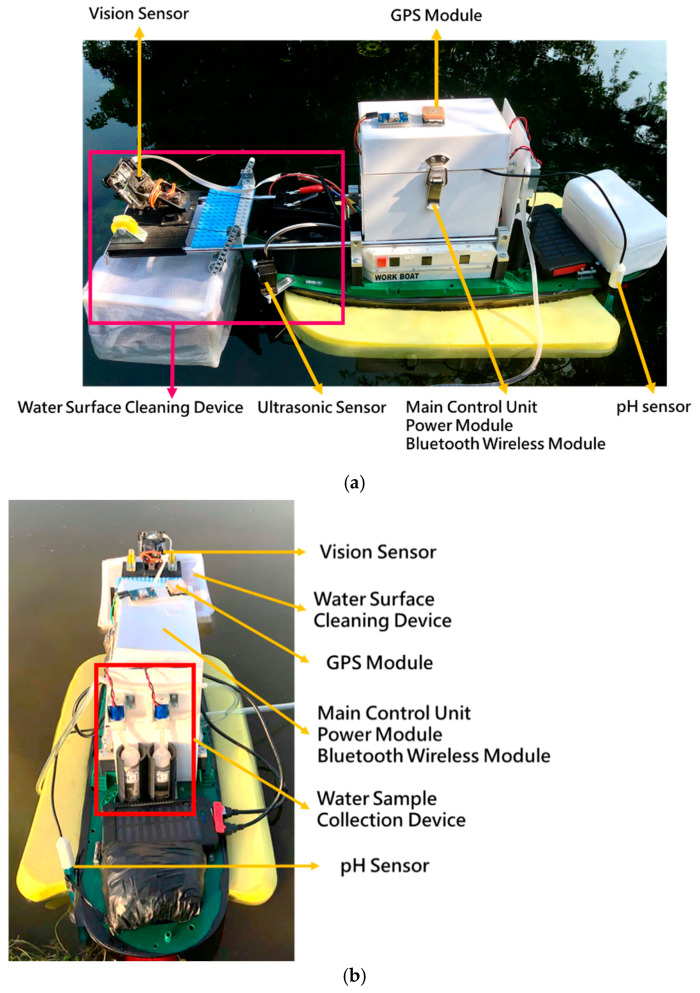
View of the MF-USV. (**a**) Side view. (**b**) Rear view.

The MF-USV was designed to avoid the obstacles, monitor the water quality, and clean the floating garbage. Field experiments were conducted on 15–18 January 2019 in the shallow Chia-Ming Lake, Taichung, Taiwan, to evaluate the performance of the MF-USV. The tests were divided into four parts: obstacle avoidance, water quality monitoring, water surface cleaning, and autonomous navigation. These tasks could be performed separately or simultaneously. Figure 15 shows the boat body of the MF-USV which is composed of the main control unit, locomotion module, positioning module (GPS module), obstacle avoidance module (ultrasonic sensors), water quality monitoring system (pH sensor and water sample collection device), water surface cleaning system (vision sensor and water surface cleaning device), communication module (Bluetooth wireless module), power module, and remote human–machine interface.

### 5.1. Obstacle Avoidance

**Figure 16:**
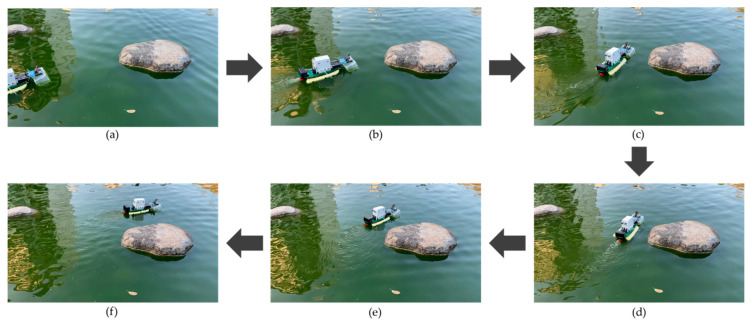
Experimental results for the obstacle avoidance task. (**a**) When ($D_{L}>$ 65 cm) and ($D_{R}>$ 65 cm), the MF-USV goes straight ahead. (**b**) When ($D_{L}$ > 65 cm) and (10 cm < $D_{R}$ ≤ 65 cm), there is an obstacle on the right front side of the vehicle. (**c**) The MF-USV turns left. (**d**) The MF-USV keeps turning left. (**e**) When ($D_{L}>$ 65 cm) and ($D_{R}>$ 65 cm), the MF-USV goes straight ahead. (**f**) The MF-USV keeps going straight ahead.

The test was conducted to check the capability of the obstacle avoidance of the MF-USV. The obstacle avoidance module of the MF-USV composed of two HC-SR04 ultrasonic sensors is designed for detecting and avoiding obstacles. Once the distances measured by the ultrasonic sensors are greater or less than the threshold values, the MF-USV executes a threshold-based obstacle avoidance algorithm to avoid obstacle collision. When ($D_{L}>$ 65 cm) and ($D_{R}>$ 65 cm), the threshold-based obstacle avoidance algorithm determines that there is no any obstacle on the front of the vehicle, and then the MF-USV goes straight ahead, as shown in Figure 16a. Subsequently, when ($D_{L}$ > 65 cm) and (10 cm < $D_{R}$ ≤ 65 cm), the threshold-based obstacle avoidance algorithm determines that there is an obstacle on the right front side of the vehicle, as shown in Figure 16b. Then, the MF-USV drives the servo motor (MG996R) in the locomotion module to control the rudder to turn left, as shown in Figure 16c,d. Once the MF-USV has avoided the obstacle successfully, the distances are measured as ($D_{L}>$ 65 cm) and ($D_{R}>$ 65 cm), the threshold-based obstacle avoidance algorithm determines that there is no any obstacle in front of the vehicle, and then the MF-USV goes straight ahead, as shown in Figure 16e,f.

### 5.2. Water Quality Monitoring

**Figure 17:**
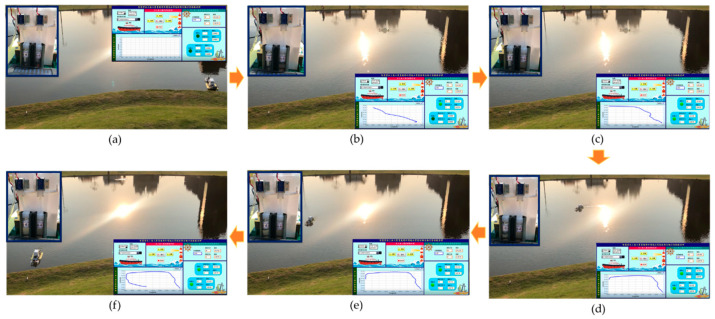
Sailing movement trajectory of the MF-USV. (**a**) The MF-USV was located at the start point. (**b**) When the water pH value at the first location was higher than 8.5 or lower than 6.5, the water was sampled by the water pump. (**c**) The MF-USV leaved the present water sampling point. (**d**) The MF-USV went ahead to search the next contaminated water point. (**e**) The contaminated water field at the second location was detected by the pH sensor and sampled by the water pump. (**f**) The water samples were conveyed to the initial location.

**Figure 18:**
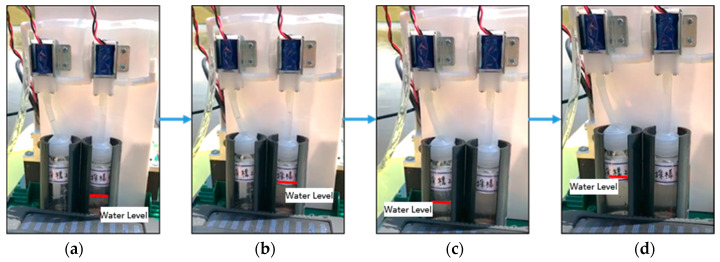
Experimental results for the water quality monitoring task. (**a**) The initial water level of the first sampling bottle. (**b**) The water level of the first sampling bottle after the first water sampling. (**c**) The initial water level of the second sampling bottle. (**d**) The water level of the second sampling bottle after the second water sampling.

The test was conducted to check the capability of water quality monitoring and water sample collection by the MF-USV. The water quality monitoring system of the MF-USV composed of the pH sensor, GPS module, and water sample collection device is designed for monitoring water quality and collecting water samples. The pH sensor and GPS module mounted on the MF-USV were used to detect the pH values of the water and position coordinates during sailing in real time, respectively. When the water quality was normal (6.5 ≤ pH ≤ 8.5), the MF-USV can automatically or manually sail on the lake and the sailing position coordinates measured by the GPS module was recorded and sent to the remote human–machine interface through the Bluetooth wireless module, as shown in Figure 17. When the water pH value at the first location (as shown in Figure 17b) was higher than 8.5 or lower than 6.5, which represented that the present water field was contaminated and the water pump was driven to extract the water for cleaning residual liquid in the pumping pipe. Then, the water on the present position was sampled by the water pump through the pumping pipe and the first electromagnetic valve was turned on to collect the sampling water in the first sampling bottle. The water level of the first sampling bottle was increased from Figure 18a to Figure 18b. In addition, the position coordinate of the present water sampling point measured by the GPS module was simultaneously recorded and sent to the remote human–machine interface. Finally, the water pump and first electromagnetic valve were turned off to finish the first water sampling task. The MF-USV leaved the present water sampling point and went ahead to search the next contaminated water point, as shown in Figure 17c,d. Once the contaminated water field at the second location (as shown in Figure 17e) was detected by the pH sensor, whose pH value was higher than 8.5 or lower than 6.5, the residual liquid in the pumping pipe was cleaned by the water extracted by the water pump. Subsequently, the water on the second water sampling position was sampled by the water pump through the pumping pipe and the second electromagnetic valve was turned on to collect the sampling water in the second sampling bottle. The water level of the second sampling bottle was increased from Figure 18c to Figure 18d. Additionally, the position coordinate of the second water sampling point measured by the GPS module was simultaneously recorded and sent to the remote human–machine interface. Finally, the water pump and second electromagnetic valve were turned off to finish the second water sampling task. After the water quality monitoring task was completed, the water samples were conveyed to the initial location and later analyzed in specialized laboratories, as shown in Figure 17f.

### 5.3. Water Surface Cleaning

**Figure 19:**
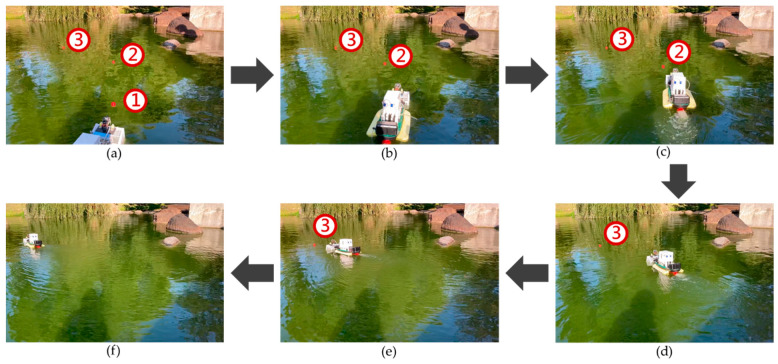
Experimental results for the water surface cleaning task. (**a**) When the first floating garbage is on the left front side of the vehicle, the MF-USV turns left about 15° for tracking the first floating garbage. (**b**) The first floating garbage is collected by the water surface cleaning device. (**c**) When the second floating garbage is on the left front side of the vehicle, the MF-USV turns left about 15° for tracking the second floating garbage. (**d**) The second floating garbage is collected by the water surface cleaning device. (**e**) When the third floating garbage is on the left side of the vehicle, the MF-USV turns left about 30° for tracking the third floating garbage. (**f**) The third floating garbage is collected by the water surface cleaning device.

The test was conducted to check the capability of the floating garbage detection and water surface cleaning of the MF-USV. The water surface cleaning system of the MF-USV composed of the vision sensor and water surface cleaning device is designed for detecting and cleaning the floating garbage. The vision sensor and its hue-based color filtering algorithm were used to detect the floating garbage on water surface in real time. When the floating garbage was detected, the MF-USV can automatically track and collect the floating garbage by using the water surface cleaning algorithm. When the vision sensor mounted on the front of the MF-USV detected the first floating garbage, which is on the left front side of the vehicle, the MF-USV could drive the servo motor (MG996R) in the locomotion module to control the rudder to turn left about 15° for tracking the first floating garbage, as shown in Figure 19a. Subsequently, the water surface cleaning device turned on the DC gear motors to control the salvage net for collecting the first floating garbage, as shown in Figure 19b. Then, the vision sensor mounted on the MF-USV detected the second floating garbage on the left front side of the vehicle, the MF-USV could drive the servo motor (MG996R) in the locomotion module to control the rudder to turn left about 15° for tracking the second floating garbage, as shown in Figure 19c. Subsequently, the water surface cleaning device was used to collect the second floating garbage, as shown in Figure 19d. Then, the vision sensor mounted on the MF-USV detected the third floating garbage on the left side of the vehicle, the MF-USV could drive the servo motor (MG996R) in the locomotion module to control the rudder to turn left about 30° for tracking the third floating garbage, as shown in Figure 19e. Finally, the third floating garbage was collected by the water surface cleaning device, as shown in Figure 19f.

Table 4 shows the success rates of floating garbage detection by the Pixy CMUcam5 vision sensor with its hue-based color filtering algorithm. When the floating garbage located in the visual angle ranged from –30° to 30° on the front of the MF-USV and the distances between the floating garbage and the MF-USV were 40 and 70 cm, the success rates of floating garbage detection were all 100%. When the floating garbage was located in front of the MF-USV (visual angle = 0°), we obtained better success rates regardless the distance between the floating garbage and the MF-USV. On the other hand, worse success rates were obtained when the floating garbage was located in a larger visual angle and at a longer distance from the front of the MF-USV. Table 5 shows the success rates of floating garbage collection when the distance between the floating garbage and the MF-USV was 130 cm. When the floating garbage was located on the left side (15°~30°), left front side (0°~15°), front side (0°), right front side (0°~15°), and the right side (15°~30°), the success rates of the floating garbage collection were 70%, 92%, 95%, 95%, and 75%, respectively.

### 5.4. Multi-Function Tests

**Figure 20:**
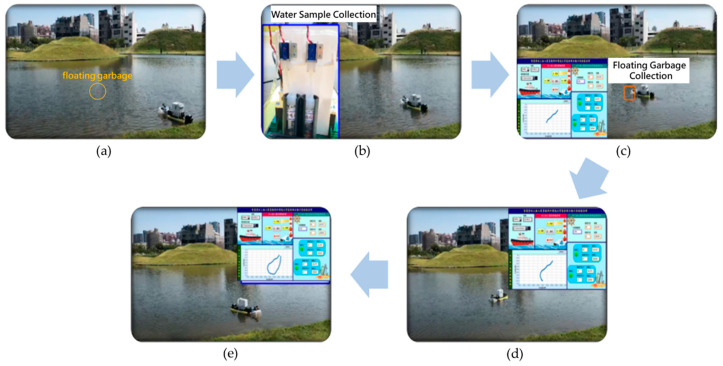
Experimental results for the multi-function tasks of the MF-USV. (**a**) The MF-USV sailed on the lake automatically and executed the obstacle avoidance task. (**b**) When the quality of water was abnormal, the MF-USV could collect the water sample in the water bottle. (**c**) When the floating garbage was detected, the MF-USV could collect and clean the floating garbage. (**d**) The MF-USV executed the obstacle avoidance task. (**e**) The MF-USV returned to the initial location automatically.

The test was conducted to check the capability of the obstacle avoidance, water quality monitoring, and water surface cleaning of the MF-USV simultaneously. At first, the MF-USV sailed on the lake automatically and executed the obstacle avoidance task by using the threshold-based obstacle avoidance algorithm, as shown in Figure 20a. Subsequently, when the quality of water was abnormal, the MF-USV could collect the water sample in the water bottle and record the position coordinate at the present water sampling point, as shown in Figure 20b. Then, when the floating garbage was detected, the MF-USV could collect and clean the floating garbage by using the water surface cleaning algorithm, as shown in Figure 20c. Finally, after the water quality monitoring and water surface cleaning tasks were completed, the MF-USV could execute the obstacle avoidance task and return to the initial location automatically, as shown in Figure 20d,e.

## 6. Conclusions

In this paper, a MF-USV has been creatively developed to avoid obstacles, collect contaminated water samples, and clean garbage floating on the water surface. The architecture of the proposed MF-USV comprises the main control unit, locomotion module, positioning module, obstacle avoidance module, water quality monitoring system, water surface cleaning system, communication module, power module, and remote human–machine interface for the purposes of obstacle avoidance, water quality monitoring, and water surface cleaning. Our experimental results have successfully validated that the MF-USV can successfully execute obstacle avoidance, water quality monitoring, and water surface cleaning tasks. Once the contaminated water field is detected, the MF-USV can collect the water sample for further analysis and send the pH value and GPS coordinates of the water sampling point to the remote human–machine interface for recording and displaying. On the other hand, when the floating garbage is detected, the MF-USV can automatically track and collect the floating garbage through the water surface cleaning algorithm. The success rates of floating garbage detection are all 100% when the floating garbage located in the visual angle ranged from −30° to 30° on the front of the MF-USV and the distances between the floating garbage and the MF-USV were 40 and 70 cm. When the distance between the floating garbage and the MF-USV was 130 cm, the success rates of the floating garbage collection were from 70% to 95% in different directions. Based on the above experimental results, we believe that the proposed MF-USV and its associated algorithms will provide a novel and effective contribution to unmanned surface vehicle design. The main contribution of this paper is to develop a low cost USV with multi-functions and its threshold-based algorithms for providing an effective tool for obstacle avoidance, water quality monitoring, and water surface cleaning tasks.

In the future, the MF-USV will be tested in more complicated field environments to develop more robust and stable methodology for water quality monitoring and water surface cleaning. Moreover, extra sensors and algorithms will be added to the MF-USV to improve the functions of obstacle avoidance, water quality monitoring, and water surface cleaning.

## Figures and Tables

**Table 1:** Function comparison of the proposed multi-function unmanned surface vehicle (MF-USV) with some existing USVs/autonomous surface vehicles (ASVs).

Reference	Function	Sensor	Algorithm
Villa et al. [5]	Obstacle avoidance	LiDAR	LOS-based GNC Algorithm
Wang et al. [8]	Obstacle avoidance	Navigation radar	IACO Algorithm
Kim et al. [9]	Obstacle detection	Vision sensors	Skip-ENet Algorithm
Steccanella et al. [10]	Waterline and obstacle detection	Camera	U-net CNN Algorithm
Guardeño et al. [11]	Obstacle avoidance	LiDAR	ATESOA algorithm
Li et al. [12]	Obstacle avoidance	Microwave radar, 4G Camera	--
	Water quality monitoring	Chl-a turbidity, water dissolved oxygen, water conductivity, oxidation-reduction potential, water temperature, salinity, pH sensors	--
Ferri et al. [6]	Obstacle avoidance	Laser scanner, Sonar, Altimeter	VFH^+^ algorithm
	Water quality monitoring	Chemical sensors (Hg, Cr, Cd, and dispersed oil)	--
Madeo et al. [13]	Water quality monitoring	pH sensor, ORP sensor, salinity sensor, dissolved oxygen probe	--
Cao et al. [14]	Water quality monitoring	pH sensor, TDS sensor, turbidity sensor	Ensemble learning algorithm
Cryer et al. [15]	Water quality monitoring	CTD, DO, pH, pCO2, nitrate, chlorophyll fluorescence, CDOM, turbidity, DOC sensors	--
Kong et al. [7]	Water surface cleaning	Vision module, Grasper	YOLOv3 algorithm
Wang et al. [23]	Water surface cleaning	Conveyor belt	--
Ruangpayoongsak et al. [24]	Water surface cleaning	Camera, waste scooper	--
Li et al. [25]	Water surface cleaning	Camera, collection box	YOLOv3 algorithm
Proposed MF-USV	Obstacle avoidance	Ultrasonic Sensors	Threshold-based obstacle avoidance algorithm
	Water quality monitoring	pH sensor	--
	Water surface cleaning	Vision sensor, salvage net	Hue-based color filtering algorithm

**Table 2:** Components of the MF-USV with their weight and power consumption.

Components	Description	Weight (g)	Estimate of the Average Electric Power Consumption (W)	Notes
Main Controller	2 × ATmega2560 (Arduino Mega2560) AVR® 8-Bit Microcontroller at 16 MHz Communication interface: Universal Asynchronous Receiver Transmitter (UART), Inter-Integrated Circuit (I^2^C), Serial Peripheral Interface (SPI), General Purpose Input/Output (GPIO)	74	0.4	
Locomotion Module	Propulsion: HMS-RF240 DC motor Rudder motor: MG996R Servo motor Motor drive module: KEYES L298N Electric propeller Rudder and actuation mechanism	135	5.52	Maximum propeller speed: 15 cm/s Maximum rudder speed: 300°/s
Positioning Module	GY-GPS6MV2 NEO-6M-5Hz GPS Antenna GPS	18	0.14	
Ultrasonic Sensor	2 × HC-SR04	17	0.02	Microcontroller collects data from the ultrasonic sensors, execute the obstacle detection and avoidance algorithm
pH Sensor	SEN0161 pH sampling probe	75	0.2	
Water Sample Collection Device	R385 Water pump 2 × Electromagnetic valve 2 × Sampling bottle	387	1.7	Total volume of two sampling bottles: 60 ml
Vision Sensor	Pixy CMUcam5 Processor: NXP LPC4330 at 204 MHz Communication interface: UART, I^2^C, SPI, USB Image sensor: Omnivision OV9715 2 × GS-9018 Servo motor	27	0.7	
Water Surface Cleaning Device	2 × HN-35GBE-1640Y DC gear motor Motor drive module: KEYES L298N Cleaning device: Salvage net	1159	0.38	
Communication Module	2×HC-08 Bluetooth wireless module	7	0.09	
Cabling and Boxes	Plastic boxes with waterproof connectors and glands	233	--	
Hulls and Deck		1073	--	
	**Total**	**3205** **(without batteries)**	**9.15**	

**Table 3:** Parameters of sensors mounted on the MF-USV.

Sensor Type	Image	Range	Working Voltage (V)	Accuracy	Producer
GPS	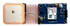	≥−161 dBm	3.3~5.0	Position: 2.5 m Velocity: 0.1 m/s Heading: 0.5°	U-BLOX ^1^
Ultrasonic	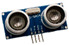	2~400 cm	3.3~5.0	0.3 cm	ELECFREAKS ^2^
pH	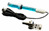	0~14	5.0	±0.1 pH	DFROBOT ^3^
Vision	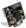	Lens field of view: ±37.5° horizontal ±23.5° vertical	6.0~10.0	Resolution: 1280 × 800 dpi	PIXYCAM ^4^

Note: ^1^ Thalwil, Switzerland; ^2^ Shenzhen, China; ^3^ Shanghai, China; ^4^ Texas, USA.

**Table 4:** The success rates of floating garbage detection by the Pixy CMUcam5 vision sensor.

	Angle	−30°	−15°	0°	15°	30°
Distance						
40 cm		100%	100%	100%	100%	100%
70 cm		100%	100%	100%	100%	100%
100 cm		94%	97%	99%	99%	96%
130 cm		89%	94%	98%	97%	93%

**Table 5:** The success rates of floating garbage collection for the water surface cleaning task.

	Angle	Left 15°~30°	Left 0°~15°	Front 0°	Right 0°~15°	Right 15°~30°
Distance						
130 cm		70%	92%	95%	95%	75%

## Data Availability

The data presented in this study are available on request from the corresponding author. The data are not publicly available due to further study will be carried out using the same data.
